# Supplementary material for: Are carbon emissions trading and green financial instruments synergistic? -Comprehensive quantitative research based on content analysis
Source: PLoS One. 2024 Mar 7;19(3):e0298601. doi: 10.1371/journal.pone.0298601 (PMC10919601; doi:10.1371/journal.pone.0298601)
Supplement: S1 Data — (ZIP) [file pone.0298601.s001.zip › Data/地级市Z_F工作报告.zip!/╣π╬≈/▒▒║ú╩╨/└┤╫╘╣1⁄2╓┌║┼í╛┬φ┐╦╩2╛▌═°í┐_2016.docx]

各位代表：

现在，我代表市第十四届人民政府，向大会作政府工作报告，请予审议，并请市政协委员和其他列席人员提出意见。

一、过去五年的工作回顾

过去五年，是北海经济社会高速发展、阔步跨越的五年。在自治区党委、政府和市委的正确领导下，我们全面贯彻落实党的十八大和习近平总书记系列重要讲话精神，坚持“真抓实干、把事干成、造福百姓”理念，抢抓机遇、砥砺奋进，夺取了三年跨越发展工程的全面胜利，走出了一条“产业带动、文化引领、开放推动”的发展新路子，完成了市十四届人大历次会议确定的目标任务，开创了北海改革开放和科学发展的新局面，提升了北海在北部湾经济区和广西发展格局中的地位。

五年来，北海经济实现跨越发展，综合实力显著增强，地区生产总值、财政收入、规模以上工业总产值等主要经济指标翻一番以上；优势产业迅猛发展，电子信息、石油化工和临港新材料三大主导产业总产值增长近8倍，电子信息产业成为北海首个千亿元产业，广西最大的外商投资项目北海林纸一体化建成投产；高新技术产业园区升级为国家高新技术产业开发区，出口加工区B区建成运行，海洋产业科技园区签约入驻科研机构19家；银滩大道、广东路和上海路跨铁路立交桥等建成通车，“七纵七横”城市主干路网基本贯通；北海机场年旅客吞吐量突破并保持百万人次，南北高铁开通运营，玉林至铁山港铁路和高速公路建成通车，合湛铁路、贵合高速公路开工建设；园艺博览园建成开园，北海滨海国家湿地公园正式获批建设；用于民生领域的财政支出占公共财政支出75%以上，完成为民办实事项目53项；新增2所本科院校，北海中学新校区、北师大北海附中、市委党校新校区建成启用；成功举办第24届世客会、第四届广西园博会、2016年世界海洋日暨全国海洋宣传日主场活动、第八届中国生态文化高峰论坛等。

五年来的主要工作和特点：

（一）坚持发展第一要务，综合实力显著提升

坚持以经济工作为主线，推动重大项目建设，发展壮大优势产业，经济规模和综合实力大幅提升，升级进位明显，经济总量由全区第十位升至第八位，财政收入由第十位升至第五位，规模以上工业总产值由第十一位升至第五位。2015年实现地区生产总值892.1亿元，是2010年的2.2倍，年均增长15.4%，增速从2012年起连续4年排全区第一；财政收入142.98亿元，是2010年的3倍，年均增长24.9%；规模以上工业总产值1871.4亿元，是2010年的6.1倍，年均增长38.6%；固定资产投资920.4亿元，年均增长20.6%；全社会用电量49.8亿千瓦时，年均增长18.5%。非公有制经济快速发展，成为推动经济跨越发展的重要力量。

（二）大力发展优势产业，经济支撑更加牢固

坚持加快发展优势产业，不断扩大产业规模，第二产业成为推动经济发展的主动力，进入了工业化中期阶段。三次产业结构由22:42:36调整为18:50:32，工业化率由1.66提高到2.52。

三大主导产业迅猛发展。坚持以“四定”办法推进重大产业项目建设，中石化北海炼化异地改造、诚德新材料、林纸一体化、中石化广西LNG、德昌电机、冠捷科技、朗科科技、三诺电子、惠科电子等项目建成投产，中电北部湾信息港、银河城市科技产业城等项目加快推进。电子信息、石油化工、临港新材料三大主导产业2015年共完成产值1582.6亿元，年均增长52.7%，中石化北海炼化成为全市税收贡献最大的企业，电子信息产业从不足百亿元发展到千亿元、占广西的半壁江山。园区的产业集聚效应更加明显，2015年园区实现规模以上工业总产值1761.7亿元，年均增长46.8%，占全市的94.1%。

现代服务业加快发展。以旅游业为龙头的现代服务业加快发展，第三产业增加值年均增长8.6%。北海入选首批国家全域旅游示范区创建城市，荣获“最美中国旅游目的地城市”称号，五年累计接待游客7902.3万人次、旅游总收入738.9亿元，年均分别增长17.9%、26.6%。银滩和涠洲岛景区提升改造初见成效，北海老城、园博园、金海湾红树林、冠山海被评为4A级景区。商贸流通业加快发展，2015年社会消费品零售总额202.9亿元，年均增长13.4%。拓展企业投融资渠道，新增主板上市企业1家、新三板挂牌企业3家。落实房地产去库存政策，房地产业平稳有序发展。

现代农业稳步发展。农林牧渔业增加值年均增长3.6%。积极创建现代特色农业示范区和“双高”糖料蔗基地，建成特色农业示范基地39个、高效设施农业15万亩、高标准基本农田45万亩，发展农业产业化龙头企业41家，完成“万元增收工程”水产示范养殖面积23.8万亩，农产品与市场衔接体系加快构建，形成合浦豇豆等特色品牌，林下经济、观光农业成效显现，北海获评国家级出口食品农产品质量安全示范区。实施农田水利建设三年攻坚，建成标准化海河堤85.9公里，在全区率先完成中小型水库除险加固任务。抗击超强台风“威马逊”取得全面胜利。

（三）加快城乡建设步伐，城市功能不断完善

统筹协调城乡发展，城市规划建设管理水平不断提高。完成《北海市城市总体规划（2013－2030）》修编，城市发展空间布局不断优化。城市建成区面积扩展到87.6平方公里，常住人口城镇化率提高到55.3%。金海岸大道、重庆路等一批道路建成通车，城市主干道路“梗阻”基本消除。完成改造小街小巷260条，惠及居民1.5万多户、7万余人。完成内涝整治二期工程，建成铁路北侧排水明渠东段和西段，内涝点从96个减少到6个，主城区内涝问题有效缓解。新建扩建110千伏以上变电站10座，开工建设500千伏变电站，供电能力大幅提升。农村基础设施建设持续加强，千里乡村公路畅通工程、农村电网改造工程成效显著，在全区率先实现行政村公路硬底化“村村通”。大力整治“四车”乱象，加强治理占道经营和环境卫生脏乱差问题，综合整治铁路沿线环境，城市交通秩序和市容市貌明显改善。

（四）深化改革扩大开放，发展活力明显增强

全面深化各项改革，推进面向东盟及粤港澳台、西南中南的开放合作，主动融入北部湾经济区同城化，改革开放取得新成效。

改革创新加快推进。扎实推进供给侧结构性改革，持续深化行政审批制度、商事制度、财税金融、国资国企、农村综合配套等方面改革。有效推进“放管服”改革，行政审批事项精简率达59%，在全区率先实行建设工程项目优化审批流程改革、行政审批中介服务去行政化和“三证合一”全覆盖。加快国税、地税征管体制改革，如期完成营改增试点改革任务。扎实推进沿边金融综合改革和农村金融改革，多项主要业务实现创新发展，银行业机构不良贷款率从两位数下降至3%以下。加快推进市属国企负责人薪酬管理、党政机关与所属企业脱钩等改革。

开放合作不断深化。积极参与共建21世纪海上丝绸之路，面向东盟和粤港澳台的开放合作不断深化。北海港口岸扩大开放获国务院批复并即将完成初步验收，铁山港1-6#泊位投入使用或基本建成，铁山港航道疏浚二期工程完成、三期工程开工建设。与北部湾经济区各市合作不断深化，通信、金融服务、户籍、交通、口岸通关、人力资源和社会保障等领域同城化取得突破性进展。外向型经济加快发展，累计招商引资实际到位资金1751.6亿元，累计全口径实际利用外资16.1亿美元，外贸进出口额年均增长22%。对外交流不断加强，文化、教育等领域的交往持续开展，《碧海丝路》赴东南亚、欧洲巡演广受赞誉。

（五）加强生态文明建设，环境质量保持良好

坚持产业发展与生态保护并举，努力保持北海良好的生态环境。环保基础设施加快完善，新建扩建污水处理厂11座、截污管网457.6公里，城市建成区生活污水处理率达90%，主城区生活垃圾无害化处理率达100%。节能减排扎实推进，单位GDP能耗累计下降15.6%，全面完成国家和自治区下达的节能减排目标任务。严肃查处和整改中央环境保护督察组交办的群众举报环境污染问题30批167件，完成北海滨海国家湿地公园部分生态修复与恢复工程。生态环境保持良好，环境空气质量优良率稳居全国前列，地表水环境质量基本达到三类标准，城市饮用水水质达标率连续五年保持100%，近岸海域水质总体为优，森林覆盖率和森林蓄积实现双增长。“美丽北海”清洁乡村、生态乡村建设深入推进，农村人居环境不断改善。先后荣获国家园林城市、国家节水型城市、国家级海洋生态文明建设示范区等称号。

（六）努力保障改善民生，发展成果充分共享

扎实做好保障和改善民生工作，不断让群众得到实实在在的好处。全市财政对民生领域的投入达393.4亿元，年均增长17.6%。城镇居民人均可支配收入27729元、年均增长10.5%，农村居民人均可支配收入9923元、年均增长12.8%。

脱贫攻坚扎实推进。加强基础设施扶贫项目建设、产业扶贫项目开发和“雨露计划”扶贫培训，累计脱贫11.4万人，39个贫困村整村推进扶贫开发全部达标，如期完成“十二五”扶贫开发任务。全面推进精准扶贫，落实精准帮扶措施，积极筹措扶贫资金，大力推进贫困村基础设施建设，脱贫攻坚取得初步成效。

社会事业加快发展。精神文明创建活动成效明显，成为“全国文明城市”提名城市。科技研发经费年均增长10%以上，科技贡献率逐年提高。五年投入93亿元打造教育“升级版”，新建、迁建中小学校、幼儿园35所，改扩建323所，中心城区中小学“大班额”问题基本解决，与北京八中、北师大附中合作办学，借智办学成效明显。高等教育和职业教育规模持续扩大，桂电海洋信息工程学院建成招生，北海艺术设计学院升格为本科院校。公共文化服务体系加快完善，全市一半以上的行政村建成村级公共服务中心，“海上丝绸之路”联合申遗扎实推进，碧海丝路大剧院投入运营。全民健身活动广泛开展，竞技体育成绩显著，足球冬训基地规模不断扩大。医疗卫生服务水平稳步提高，开工建设卫生基建项目74个，全市医院床位增加近1倍，市中医医院综合大楼等一批医疗设施建成使用，我国首支国家级海（水）上紧急医学救援队伍落户北海。落实全面二孩政策，促进人口均衡发展。外事、侨务、民族、宗教、档案、新闻出版、地方志、妇女儿童、残疾人等方面工作取得新进展。

社会保障持续完善。覆盖城乡的社会保障体系基本形成，社会保险制度实现全覆盖。社会保险待遇显著提高，市辖区城市低保标准每人每年提高到5760元，农村低保标准每人每年提高到3000元，职工医疗保险的最高支付限额提高到25.6万元，大病保险实现全覆盖。累计开工建设保障性住房和棚户区改造32168套(户)，建成24954套(户)，21771个住房困难家庭喜迁新居，改造农村危房19601户。实施农村饮水工程153处，解决农村34.5万人的饮水安全问题，农村自来水普及率达75%、居全区前列。完成水库移民新村建设工程125个、移民旧房改造2540户。城镇就业、农村劳动力转移就业分别新增17.1万人和7.9万人，2015年城镇登记失业率3.14%、低于年度控制数0.86个百分点。

社会治理不断加强。全面推进依法治市，公共法律服务体系建设工作走在全区前列，荣获全国“六五”普法先进市。社会治安防控体系建设进一步加强，完成平安二期工程，建成社会治安监控、智能交通管理系统，实现城区重点区域和主要交通道路全覆盖。加快推进应急管理体系建设，有效预防和处置突发事件。加强矛盾调处和信访工作，妥善化解社会矛盾。强化公共安全监管，安全生产、食品药品安全监管成效明显。国防动员和后备力量建设、海防、人防和双拥共建等工作取得新成效。

各位代表！

五年来，我们认真落实市委常委会“三重一大”集体决策制度，重要工作事项及时向市委请示报告。贯彻执行市人大及其常委会各项决议决定，定期向市人大及其常委会报告工作、向市政协通报情况，自觉接受市人大的法律监督、工作监督和市政协的民主监督，人大代表建议、政协提案办结率和满意率均达100%。坚持依法行政，建设法治政府，在全区率先成立政府法律顾问团，大力推进政务公开和政府信息公开工作。努力优化政务服务，在全区率先实现政务服务体系全覆盖，群众对政务服务中心办事保持高满意度。落实全面从严治党“两个责任”，推进党风廉政建设和反腐败斗争，严格执行中央八项规定，加强作风建设，深入开展查处发生在群众身边的“四风”和腐败问题专项工作，加强行政监察和审计监督，严肃查处并公开曝光一批违纪违法案件。

今年以来，在经济下行压力持续加大的情况下，我们统筹做好稳增长、促改革、调结构、惠民生、防风险各项工作，全市经济保持平稳较快增长，实现了“十三五”的良好开局。

各位代表！

五年来，我们咬定产业发展不放松，狠抓重大项目不动摇，坚持改革开放不停步，推进城乡建设不懈怠，增进民生福祉不含糊，加强自身建设不马虎，全市经济更加繁荣、城市更加宜居、社会更加和谐、人民更加幸福。这些成绩的取得，是自治区党委、政府和市委正确领导的结果，是全市广大干部群众真抓实干、团结拼搏的结果，是历届领导班子和历任领导长期努力打下良好基础的结果，是市人大、市政协大力支持、有效监督的结果，是各民主党派、各人民团体、各界人士和驻市单位、驻市部队、武警官兵共同努力的结果。在此，我谨代表市第十四届人民政府，向所有关心、支持、参与北海建设，为北海发展付出心血和汗水的同志们、朋友们表示衷心的感谢，致以崇高的敬意！

在肯定成绩的同时，我们也清醒地认识到，经济社会发展和政府工作还存在不少困难和问题，主要表现在：主导产业的产业链延伸不够、配套不完善，第三产业发展相对缓慢，产业结构有待调整优化；重大产业项目储备不足，创新能力不够强，新产业、新业态和新模式发展不快；城市建设管理亟需提升水平，公共服务设施“短板”仍较突出；政府系统一些工作人员主观能动性差，执行力不强，行政效率不高，办事不按程序甚至不守规矩的现象仍时有发生。对于这些问题，我们一定要保持朝乾夕惕之心，恪尽职守，有所作为，孜孜不倦加以克服和解决。

二、今后五年的主要任务

中国共产党北海市第十一次代表大会描绘了北海宏伟的发展蓝图，确定了今后五年工作的指导思想和发展的总体目标，对经济社会发展作出了全面部署。

未来五年，北海处于在广西率先全面建成小康社会的决胜阶段，处于全区基本建成国际通道、战略支点、重要门户的关键时期，处于新型工业化、城镇化加速发展和经济转型升级的重要关口，机遇和挑战并存。随着“一带一路”战略深入实施、广西对外开放步伐加快、北部湾经济区升级发展加速、供给侧结构性改革力度加大，北海发展的新机遇和新动能不断增加，三大产业的带动作用和集聚效应不断呈现，干部群众推动北海加快发展的责任感和使命感不断增强，经济社会发展具有良好的基础和许多积极因素，但也面临不少困难和问题，经济下行压力仍然较大。我们必须主动适应经济发展新常态，审时度势、积极应对，凝心聚力、乘胜前进，奋力谱写北海改革开放和科学发展新篇章。

今后五年政府工作的总体思路是：高举中国特色社会主义伟大旗帜，以马列主义、毛泽东思想、邓小平理论、“三个代表”重要思想、科学发展观为指导，深入学习贯彻习近平总书记系列重要讲话精神，坚持以人民为中心的发展思想，遵循创新、协调、绿色、开放、共享的发展理念，推进“五位一体”总体布局和“四个全面”战略布局，贯彻落实市第十一次党代会的决策部署，加快新型工业化和城镇化进程，实施“四大会战”，发展优势产业，提升城市功能，持续改善民生，再创开放发展新优势，努力把北海建设成为充满生机与活力的创新型开放城市。

今后五年全市经济社会发展的总体目标是：实现高于全国全区的经济发展速度，在广西率先全面建成小康社会，为广西构建“国际通道、战略支点、重要门户”发挥重要作用，把北海建设成为创新型开放城市。主要经济指标保持较快速度增长，地区生产总值年均增长10%、财政收入年均增长10%、规模以上工业总产值年均增长15%、固定资产投资年均增长12%、社会消费品零售总额年均增长9%、城镇居民人均可支配收入年均增长9%、农村居民人均可支配收入年均增长10%，在广西率先完成脱贫攻坚任务。推动工业化、信息化、城镇化、农业现代化同步发展，经济实力持续增强，城市功能不断完善，改革开放全面深化，文明程度显著提高，生态环境保持良好，人民生活加快改善，建成一个既有“里子”、又有“面子”，既有产业支撑、又有滨海旅游独特魅力的城市，继续在北部湾经济区发挥引领作用。

上述总体目标，符合中央和自治区对北海发展的期望和要求，符合经济发展规律和北海现阶段发展特征，与市“十三五”发展规划目标任务相衔接，与市第十一次党代会确定的目标相一致，顺应了全市人民对美好生活的向往。

我们要在“十二五”的基础上，调动一切积极因素，凝聚最大合力推动发展，以最大公约数实现、维护和发展好群众利益。要更加重视改革创新，着力增强经济发展新动力；更加重视发展壮大产业，着力增强综合实力；更加重视城市规划建设管理，着力建设全市人民的美好家园；更加重视发展海洋经济，着力培育新的经济增长点；更加重视开放合作，着力发展开放型经济；更加重视发展县域经济，着力打赢脱贫攻坚战；更加重视回应群众的关切，着力为百姓办更多的好事实事；更加重视政府自身建设，着力提高政府行政效能，确保全面完成目标任务。

今后五年政府工作的主要任务是：

（一）实施工业转型升级大会战，推动产业创新协调发展

产业是北海经济发展的核心支撑，是城市发展的动力之源。做大做强产业是发展之纲、施政之要，是抓好以发展为主题各项工作的重中之重。我们要坚持创新引领发展，推动工业化与信息化融合发展，持续发力做大做强优势产业，增强工业在经济发展中的支撑作用，协调发展三次产业，推动产业迈向中高端水平。

一是坚持创新驱动发展。把创新摆在发展全局的核心位置，实施“创新驱动助力工程”，推进大众创业、万众创新，大幅提升科技进步对经济增长的贡献率。发挥科技创新的引领作用，促进科技与产业深度融合，加大技术创新和改造力度，推动北海的传统产业和优势产业加快转型升级。实施领航人才计划和人才小高地建设提升工程，开展“特聘专家”设岗选聘工作，优化人才成长环境，培养引进一批创新创业人才。加大对创新的投入，创立和用好中小企业创新引导基金，推动创新资源向企业集聚，培育壮大一批竞争力强、产品市场前景好的创新型企业。以北海国家级科技企业孵化器为依托，发挥创业主体作用，构建“产学研用”一体化合作机制，加快建设研发、检验、检测、认证、创新创业孵化、融资租赁、应用推广等一体化服务的创新示范平台，促进科技成果转化，增强创新发展的能力。

二是持续发展壮大新型工业。以“三大工程”为抓手，实施工业转型升级大会战，加快推进新型工业化，完善“两核七园一带”工业布局，推动产城融合，发展非公经济，培育强优企业，扶持中小企业，力争到2021年工业总量比2016年翻一番。

实施产业延伸工程。立足存量企业，坚定不移做大做强电子信息、石油化工、临港新材料三大主导产业，加大支持力度、促进企业降本增效，加快完善产业链配套，引进上下游企业，发展配套产业。推动电子信息产业向智能制造迈进，大力发展电子信息制造业，培育发展软件和信息服务业、大数据产业，推进中电北部湾信息港、国家信息技术（安全）产品质量检验中心和高新区软件与信息服务业基地等建设，努力打造中西部地区重要的信息产业制造基地、面向东盟的电子产品出口基地和中国—东盟信息港重要基地，2021年力争实现产值2000亿元。推动中石化北海炼化公司项目升级改造，争取到“十三五”期末炼油综合能力达到千万吨级，加快广西LNG（二期）等项目建设，延伸上下游产业链，构建现代临港石化产业基地，2021年力争实现产值1000亿元。发挥诚德新材料的龙头带动作用，支持企业开拓市场，加快发展不锈钢冷轧薄板及制品，打造合金新材料产业基地，2021年力争实现产值1000亿元。

实施扩容增量工程。加大招商引资力度，围绕园区布局和优势产业发展策划储备一批重大项目，重点以产业链招商，积极承接产业转移，促进一批新项目尽快落地，发展一批新企业，持续推动产业扩大规模、提高质量。积极培育发展新兴产业，重点发展新一代信息技术、先进装备制造、新材料、新能源、生物医药、节能环保等产业，加快形成新产能。大力推进林纸一体化项目二期，延伸林纸一体化产业链，将林纸产业打造成为北海的优势产业。加快神华国华广投北海能源基地等能源项目建设，促进新能源产业发展。积极发展食品加工业，提高产品附加值，扩大产业规模，2021年力争实现产值200亿元。大力发展县域工业，加快推进合浦工业化进程，到2021年，力争县域工业对全县GDP的贡献率达70%左右、对财政收入的贡献率达40%左右，把合浦工业发展成为全市工业新的增长点。

实施平台建设工程。加快产业园区基础设施建设，创新完善管理体制和运行机制，提升园区服务水平，增强园区承载能力，把园区打造成为推动产业转型升级、集聚发展的重要平台。推动北海出口加工区升格为综合保税区，北海工业园区升级为国家级经济技术开发区，铁山港(临海)工业区升级为自治区级园区，北海国家高新技术产业开发区扩区扩容、建成创新型特色园区。加快建设合浦工业园区、铁山东港产业园，力争到2021年分别实现产值300亿元。加强企业服务，落实和改进扶持措施，用好产业发展扶持基金，用足小微企业扶持政策，帮助企业降低成本、提升效益，增强市场竞争力，提升企业发展能力。

三是提升发展现代服务业。推动生产性服务业与生活性服务业协调发展，促进服务业转型升级，加快发展第三产业。大力发展交通运输、现代物流、金融保险、信息和商贸服务等服务业，推动生产性服务业向专业化和价值链高端延伸。积极发展滨海旅游、商贸、文化创意、健康养老、住宿餐饮等服务业，加快生活性服务业向精细和高品质转变。推动一批满足现代城市商业新业态、新需求的大型城市综合体尽快建成运营，打造城市新商圈。规划建设一批专业市场，形成一批服务业集聚区，促进现代服务业集聚发展。综合施策，积极推动房地产去库存，促进房地产业平稳健康发展。实施电商北海工程，促进电子商务加快发展。

四是加快发展现代农业。坚守耕地红线，稳定粮食生产，加快建设高标准基本农田，改善农业生产条件，2021年农林牧渔业增加值突破200亿元，农村居民人均纯收入超过全国平均水平。建立健全农业标准化体系，创建国家农业标准化示范区，拓展现代特色农业示范区。提升农业产业化水平，积极培育家庭农场、农民专业合作社等新型农业经营主体。大力发展优良畜禽品种的养殖和加工，积极培育观光农业、生态农业、林下经济，做强合浦豇豆等特色产业，推进原料林、“双高”糖料蔗和秋冬菜基地建设。加快发展“互联网+”农业，培育发展农产品电子商务、信息农业、订单农业。盘活集体就业用地，发展农村集体经济。健全“三农”投入稳定增长机制，全面落实惠农强农政策。

（二）实施城乡建设管理大会战，全面推进新型城镇化

城市让生活更美好。我们要全面贯彻中央和自治区城市工作会议精神，落实北部湾城市群建设规划，坚持以国际视野和先进理念，高起点规划、高标准建设、高水平管理，不断提升城市环境质量、人民生活质量、城市竞争力和发展能力。

一是优化城市空间布局。全面开展城市设计，推动多规合一，落实新修编的北海市城市总体规划（2013-2030年），加快编制控制性详细规划和各项专项规划，维护规划的严肃性和权威性，新城区要重点从整体平面和立体空间上统筹城市建筑布局，老城区要重点解决环境品质下降、空间布局不合理等问题，塑造滨海特色城市建筑风貌和城市品格。实施区城共建产城联动工程，坚持以城聚产、以产兴城、产城联动，统筹推进北海组团和铁山港（龙潭）组团联动发展、北海城区与合浦县城一体化发展，加快推进银海新城、廉州湾滨海新城、铁山港新城、海洋产业科技园区开发建设，加快建设市区铁路以南区域，优化中心城区公共服务设施布局，促进市区人口合理分布。

二是加快完善城市功能。实施市政基础设施完善提升工程。进一步优化城区路网结构，用三年左右时间打通铁路以南区域主次干道，建成江苏路、浙江路、杭州路、长沙路、湘潭路和南京路，规划建设冯家江大桥、西村港大桥和滨海公路关井至营盘段，打通西南大道东延线，进一步拓展城市框架。提升完善电力、通信、燃气、给排水等城市基础设施，新建和改造停车场、农贸市场、环卫等一批市政公用设施。增建广场和社区小绿地，提升城市主干道、园林和排水明渠绿化水平，大力营造绿色生态环境，加快建设“国家生态园林城市”。积极发展公共交通，提高公交出行率。

实施社会事业基础设施补短板工程，建成市体育馆、图书馆新馆、博物馆、档案馆、群艺馆、工人文化宫、青少年宫、妇女儿童活动中心、老干部活动中心等公共场馆，加快建设广播电视中心和北部湾体育中心等项目，积极筹划建设文化艺术中心、科技馆等一批新的公共设施。加快建设新城区的医院和社区卫生服务中心、学校和幼儿园等民生设施。

加快推进海绵城市建设，储备和实施一批海绵城市基础设施项目，加强城市地下综合管廊建设，推广应用节水、透水、集雨技术。实施内涝整治三期工程，完善城市排涝体系，基本解决主城区内涝问题。抓紧开展冠岭公园、鲤鱼地绿心等的规划建设。

三是加强城市管理治理。着眼于创建全国文明城市，针对城镇化和户籍改革中人口流动加剧的趋势，完善城市管理工作机制和执法体制，加快智慧城市建设，推广应用大数据、物联网等新一代信息技术，不断提升城市管理法治化、规范化、精细化、信息化水平，为市民、游客和投资者提供高效便捷优质的服务。开展市容市貌综合治理，加强巡查监管，遏制和清理乱搭乱建、乱摆乱卖、乱涂乱贴现象。加强环境卫生管理，提升环卫机械化清扫率，保持城市环境干净整洁。开展城市道路交通秩序综合整治，加大力度整治“四车”和出租车的违规违法营运行为。打造城市管理示范街（区），形成城市管理新亮点。

四是推动城乡一体化发展。统筹推进城乡公共资源均衡配置，推动城市基础设施和公共服务向农村延伸，有序推进农业转移人口市民化，“十三五”期末常住人口城镇化率达60%。实施大县城战略，推进合浦县城和铁山港中心城区开发建设，加快中心镇和小城镇发展。实施农村公路安全生命防护工程，加强农村道路硬化、农田水利、饮水安全、电网改造和环卫设施等建设，加快大型水库除险加固、标准化海河堤建设，建成一批水库移民新村。

（三）实施全域旅游城市建设大会战，提升旅游业发展水平

旅游业是具有内生动力的第三产业，也是北海的优势产业。我们要按照创建“国家全域旅游示范区”的要求，实施全域旅游三年行动计划，推动旅游产业转型升级和跨越发展，到2021年，旅游接待人数、旅游总收入、入境人次和收入比2016年翻一番，努力把北海建设成为区域性国际化滨海旅游名城。

一是加快景区景点建设。推进银滩改造提升工程，加快创建5A景区，规划建设一批高端旅游酒店、休闲街区和主题游乐项目，打造高端滨海旅游度假区。加快涠洲岛整体开发，建设一批高品质、国际化的旅游项目和配套设施，打造国际高端休闲度假旅游海岛。按5A级标准提升建设北海老城、园博园等景区，加快金海湾红树林生态旅游区、北海滨海国家湿地公园等景区的提档升级，统筹推进星岛湖、曲樟湖光山水等景区开发。鼓励和支持民间博物馆群建设，将北海工艺美术博览园、合浦汉文化公园、白龙城遗址打造成文化旅游新景点。支持合浦县和涠洲岛旅游区创建广西特色旅游名县（区）,建设一批旅游名镇、名街、名村。

二是打造特色旅游产品。整合旅游资源，优化产品结构，大力发展高端旅游、特色旅游、红色旅游、乡村旅游、个性化旅游，打造海洋特色旅游品牌，培育旅游消费热点，推动旅游由传统观光型向高端休闲度假和健康养生型转变。促进旅游和文化、体育等相关产业融合发展，培育发展历史文化、康体养生、海洋运动体验等特色旅游产品。加快建设北部湾邮轮母港，开拓国际国内邮轮旅游航线，打造海上丝绸之路国际旅游精品。

三是提升旅游公共服务水平。完善城市道路交通标识和旅游景点标识体系，加快建设旅游集散中心、咨询服务中心、旅游信息服务平台、景区停车场等服务设施，增强国际化旅游服务功能。发展“互联网+”智慧旅游，提升旅游市场主体经营服务水平。加强旅游市场综合治理，建立全域旅游联合执法机制，完善旅游监管和旅客投诉快速处理机制，促进行业自律。深化区域旅游合作，努力开拓国内外客源市场，把北海建设成为吸引北方国家和地区游客的重要旅游目的地。加大旅游形象品牌宣传推介力度，鼓励社会力量举办有利于提高城市知名度、美誉度的活动，增强北海旅游的吸引力和影响力。

（四）实施农村脱贫攻坚大会战，坚决打赢脱贫攻坚战

脱贫攻坚是“十三五”时期一项极其重要的工作，责任重于泰山。我们要全面落实国家和自治区精准扶贫脱贫政策，重点支持合浦县脱贫攻坚，按照贫困户和贫困村的脱贫标准，确保到2018年全市79个贫困村脱贫摘帽、4.37万农村贫困人口脱贫销号，并力争年人均纯收入高于自治区确定的脱贫标准。

一是全面推进精准帮扶精准脱贫。按照“八有一超”贫困户脱贫标准，实施扶持发展生产、转移就业、教育扶智、医疗救助、政策兜底“五个一批”脱贫措施，因地制宜、因人施策，实现精准脱贫。统筹安排各类扶贫资金，用好用活扶贫小额信贷，组织贫困户参加农民专业合作社，扶持发展特色产业，提高经营性收入。加强对贫困劳动力的文化教育和技能培训，促进转移就业，增加务工收入。落实“雨露计划”资助政策，防止因学致贫、因学返贫。对丧失劳动能力的贫困人口落实低保政策，对因病致贫、返贫的贫困人口提供医疗救助保障。落实危房改造政策，切实解决贫困户的住房问题。

二是加快推进贫困村脱贫摘帽。按照“十一有一低于”贫困村脱贫标准，补足短板，加快脱贫摘帽。大力推进贫困村基础设施建设，优先安排和加快推进贫困村的水、电、路和环境卫生等一批项目，加快完成20户以上自然村通村道路硬底化，改善贫困村生产生活条件，为贫困村发展创造有利条件。扶持发展村级集体经济，支持贫困村建设集体经济项目，增加集体经济收入，增强村级组织带领群众脱贫致富的能力。

三是落实脱贫攻坚责任。强化党政主要领导负总责的脱贫攻坚责任制，落实市抓总协调、县区为主体、乡镇村抓落实、部门配合的工作机制，明确各级各部门和扶贫工作人员的责任。加强扶贫领域监督执纪问责，切实提高扶贫工作效率，确保扶贫资金、扶贫措施和各项工作落实到位。广泛动员社会力量参与，调动贫困户的积极性，形成强大合力，坚决打赢脱贫攻坚战。

（五）大力发展海洋经济，打造经济发展新引擎

拓展蓝色经济空间，是北海发挥优势、加快经济发展的重要途径。我们要坚持海陆统筹、规划引领、科技先行，大力发展海洋产业，培育新的经济增长点，加快建设海洋强市。

一是科学制定海洋经济发展规划。统筹海陆资源配置，完善海洋经济发展规划和海洋功能区划，优化海洋产业空间布局，提高海洋综合开发水平，积极打造现代临港产业区、邮轮经济区、现代渔港经济区、海洋战略性新兴产业区“四大核心功能区”。

二是大力发展海洋产业。做大做强临港工业、港口物流、海洋运输、粮油加工、船舶修造等临港产业，培育发展海洋工程装备制造、港口物流机械制造等产业。大力发展现代渔业，推动传统渔业向集约型、科技型转变，拓展远海、远洋作业，发展特色水产养殖，推广标准化健康养殖，建设海洋牧场。提升发展水产品精深加工，加快发展水产品仓储物流。加大力度整治地角内港、外沙内港和电建渔港，恢复渔港风貌，增强旅游功能。加快建设营盘中心渔港，推动海洋产业重大项目尽快落地。创新发展海洋信息、港航服务、海洋科技服务等海洋服务业，积极发展海洋生物医药、海洋能源、海水综合利用等海洋新兴产业。

三是提高海洋科技创新能力。加快建设北海海洋产业科技园，引入更多国家级海洋研究机构和高校科研院所，引进一批涉海科技企业，加强海洋科学研究，加快建设海洋科技企业孵化器，打造科研服务平台和产业示范基地，促进海洋科技成果产业化，提高海洋资源开发利用水平，加快打造中国南方海洋科技城。

（六）推进全面深化改革，增强城市发展新动力

深入推进体制机制创新，以供给侧结构性改革为突破口，落实“三去一降一补”措施，推进重点领域和关键环节改革，确保中央和自治区各项改革举措落地生根、开花结果，增强城市创新发展的活力和动力。

一是深化政府职能转变。持续推进简政放权、放管结合、优化服务，全面推行权力清单、责任清单、负面清单，明晰权责利关系，强化事中事后监管，提高政府公信力和行政效率。减少行政审批事项，下放审批权限，优化审批流程，提高审批效率。深化商事制度改革，全面推行“六证合一、一照一码”登记制度改革。大力推进电子政务数据交换和信息共享平台建设，逐步实现政务服务数据资源跨部门、跨区域、多层级信息共享。

二是深化财税金融改革。进一步完善政府预算体系，建立跨年度预算平衡机制，积极推进预决算公开。深化国税、地税征管体制改革，与全区同步建成现代化税收征管体制。推进沿边金融改革，拓展跨境金融业务。深化农村金融改革，加大对“三农”的金融支持。推进金融服务创新，大力发展普惠金融和绿色金融，进一步优化金融生态环境。创新投融资机制，保障市场公平，撤除不合理的准入门槛，扩大社会资本投资途径，鼓励社会资本进入公共服务和基础设施领域，推广政府与社会资本合作（PPP）模式，为社会资本提供更好的融资服务。

三是深化其他领域的改革创新。推动国资国企改革，积极发展混合所有制经济，落实减税降费、物流补贴等政策措施,有效降低企业生产经营成本。深化农村综合配套改革，加快农村土地承包经营权、集体建设用地和宅基地确权登记颁证。深化城镇户籍与住房制度改革，实施差别化户籍管理制度。深化北部湾经济区同城化改革，继续推进十大领域资源重组，最大限度促进各种资源要素在北部湾经济区自由流动。

（七）提高开放合作水平，加快发展开放型经济

开放是北海最大的比较优势，是北海最根本的历史传承。我们要充分发挥北海对外开放的优势和潜力，实施更加积极主动的开放带动战略，落实自治区“四维支撑、四沿联动”要求，深化与北部湾经济区各市的合作，努力构建开放发展新格局。

一是加快完善开放平台和通道。发挥园区在对外开放中的窗口作用，提升园区的开放合作平台功能，加强产业和技术合作，促进项目引进，创新发展加工贸易，推动北海开放发展。加快建设铁山东港产业园和北海—湛江粤桂北部湾经济合作区，打造与粤港澳台合作新平台。推进海洋合作试验区和旅游合作区建设，探索对外开放合作新模式。建成合湛铁路、贵合高速公路和铁山港进港铁路专用线、铁山港至石头埠铁路支线、铁山港至啄罗铁路支线并投入运营，加快建设合浦至铁山港铁路、广西滨海公路大风江到高德段等项目。建设北海动车所，增开城际列车、增加进出北海的动车车次，开通直达中南西南的“五定”铁路运输班列。完善国际国内航空航线网络，积极培育旅游航空市场，助力开拓商务航空市场，借力大型航空枢纽，争取把北海机场建设成为通达国内省会城市、面向国际的重要开放空港。加快铁山港以及铁山东港码头建设，完成北海铁山港航道二期扩建、三期工程和15万吨级航道工程，实现北海港口岸扩大开放，加快打造亿吨大港。

二是深化对外合作交流。落实“四维支撑”要求，突出开放发展的战略重点，培育新的开放动能和经济增长点。坚持向南开拓，服务国家周边外交战略，深化同以东盟为重点的21世纪海上丝绸之路沿线国家城市的合作，加强海上互联互通、产业合作、贸易往来、人文交流等领域合作，拓展国际友好城市交往。坚持向东开拓，面向粤港澳台寻求开放合作突破点，提升对粤港澳台开放合作水平。坚持向西向北开拓，融入北部湾经济区一体化进程，打造便捷高效的西南中南出海大通道，增强服务西南中南地区开放发展功能。坚持向发达国家开拓，对接欧美日韩等经济体先进生产力，促进发展新兴产业。推动有实力的企业“走出去”，拓展发展空间。

三是大力发展外向型经济。充分发挥科技兴贸创新基地（电子信息）、加工贸易产业梯度转移重点承接地和外贸转型升级专业型示范基地的作用，提高投资与贸易便利化水平，创新利用外资方式，扩大利用外资规模。加快发展对外贸易，调整优化进出口结构，扩大水产品、高新技术产品出口，增加先进设备和短缺资源进口。积极发展跨境电商，建设电子商务产业园。

（八）努力推进绿色发展，建设生态美丽北海

既要金山银山，又要绿水青山。我们要始终坚持绿色发展，保护好北海的生态环境，守护好北海的碧海银滩。

一是大力发展生态经济。坚持产业生态化、生态产业化、生活低碳化，从严控制高污染行业，完成节能减排目标任务。加强资源节约和综合利用，推进北海出口加工区循环化改造示范园区建设，实施一批循环经济项目，培育一批循环经济企业，加快构建比较完善的循环经济和清洁生产体系。

二是加快完善环保基础设施。按照“完善管网、雨污分流改造、控源截污、流域综合治理”原则，实施雨污分流改造整治工程，加快推进红坎污水处理厂提标改造、大冠沙污水处理厂尾水深度处理等项目，完善污水管网系统，实现主城区雨污分流或截流。规划建设再生资源产业集中区，加快建设垃圾无害化处理设施，实现垃圾收运处理企业化、市场化，推进垃圾焚烧发电项目，完善建成区生活垃圾收运处理系统，减少垃圾收运站点对周边居民生活环境的影响。

三是加强生态环境保护。全面贯彻大气、水、土壤污染防治“三个十条”，深入推进治污减排，强化环境保护执法监察。加强饮用水水源地保护和综合整治，加快实现市区地表水供水，确保饮用水安全。推进冯家江与南流江流域水生态治理，实施城市水系恢复连通工程，提升城市水生态环境。实施涠洲岛全岛垃圾分类及无害化处理工程，解决海岛供水瓶颈，推广低碳交通，确保海岛永续发展。加强自然保护区、湿地公园和珊瑚礁海洋公园的建设管理，推进海域海岛海岸带整治修复和保护，扩大海洋生态文明示范区建设成果。

四是加强城乡环境治理。加大重点行业污染治理力度，狠抓扬尘污染、建筑渣土无序倾倒等治理，规划建设建筑渣土消纳场。以农村人居环境基础设施建设为重点，大力实施改房、改水、改厕、改能、改路工程，扎实推进“美丽北海”宜居乡村、幸福乡村建设，实施生态修复，抓好乡村环境综合整治，加快创建国家级生态乡镇。积极回应群众呼声，大力整治环境污染问题。

（九）繁荣发展社会事业，让人民充分共享发展成果

保障和改善民生是政府工作的出发点和落脚点。我们要坚持共享发展，积极回应群众“贫有所恤、学有所教、病有所医、住有所居、老有所养”的共同期盼，补齐民生短板，加快发展社会事业，健全基本公共服务体系，做大“蛋糕”、分好“蛋糕”，让百姓有更多的获得感。

一是繁荣发展先进文化。坚持社会主义先进文化前进方向，积极培育和践行社会主义核心价值观。加快创建全国文明城市，深化群众性精神文明创建活动，提高文明服务水平，加强社会公德、职业道德、家庭美德和个人品德建设，树立模范典型，全面提升市民文明素质和城市文明程度。大力发展文化事业，加强公益性文化设施建设，规划建设一批群众企盼迫切、城市功能必需的大型公共文化设施。推动北海文艺精品创作，弘扬地域特色文化，加强文化遗产传承与保护，积极推进“海上丝绸之路”联合申遗，深化对外文化交流合作，加强文化市场监管。加快发展文化产业，培育发展一批重点文化企业，规划建设特色文化产业示范园区和文化产业示范基地。推进传统媒体与新兴媒体融合发展，增强主流媒体影响力和传播力。实施全民健身计划，增加街道、小区公共健身活动设施，积极开展群众体育活动，推进体育项目冬训基地建设，积极发展以足球产业为重点的体育产业。

二是优先发展教育事业。以提高教育质量为核心，推进义务教育均衡发展、高中阶段教育突破发展、现代职业教育加快发展、学前教育多元化发展、高等教育规模化特色化发展，力争在全区率先基本实现教育现代化。加强学校标准化建设，优化城乡学校布局，引进优质教育资源，促进公共教育资源配置公平。加快建设和改造一批中小学校及幼儿园，规范学生午托机构管理。

三是加快发展医疗卫生事业。贯彻新形势下卫生与健康工作方针和目标，将健康融入所有政策。优化医疗机构布局，加强基层医疗机构建设，着力提高公共卫生与基本医疗服务的能力和质量，完善全民医疗保健、城镇社区卫生服务体系和农村三级医疗卫生服务网络，方便群众看病就医，实现“健康北海”和人人享有基本医疗卫生服务的目标。加快建成广西海上紧急医学救援中心、市人民医院异地扩建一期工程、市第二人民医院整体搬迁和市儿童医院等项目，启动建设中医医院、妇幼保健院新院区。引进优质医疗资源，鼓励发展民营医疗机构，加快发展中医药健康服务。落实全面两孩政策，促进人口长期均衡发展。

四是努力提高社会保障水平。实施更加积极的创业就业政策，加强就业援助，促进城乡劳动力创业就业，构建一批创业创新公共平台，建设一批小微企业创业创新基地，加快发展“互联网+”创业网络，推进创业孵化基地建设，打造一批“创业社区”“创业街区”。推进全民参保计划，进一步完善城镇职工、城乡居民和机关事业单位养老保险制度，建立健全全民共享、公平可及的社会保障体系。以满足农业转移人口市民化的住房需求为主要出发点，购租并举，加强保障性住房建设，加快棚户区、城中村和农村危旧房改造。健全社会救助体系，发展社会福利和慈善事业，加强对社会弱势群体的帮扶救济，保障妇女儿童合法权益。积极应对人口老龄化，大力发展老龄事业。

（十）全面推进法治建设，实现社会和谐稳定

坚持党的领导、人民当家作主、全面依法治国有机统一，加快建设法治城市。落实《法治政府建设实施纲要(2015—2020年)》，深入推进依法行政，加快建设法治政府。大力开展“七五”法治宣传教育，弘扬社会主义法治精神，增强市民法治意识，推动公共法律服务体系全面建成，健全社会信用体系。

改进和创新社会治理，强化社区组织自治功能，构建全民共建共享的社会治理格局，提高社会治理法治化水平。加快完善社会治安防控体系，防范和惩治违法犯罪活动，提高人民群众安全感满意度。完善群众诉求表达机制，深入开展基层矛盾纠纷调处和信访积案化解工作，有效预防和化解各类社会矛盾。

落实安全生产“党政同责、一岗双责”，明确监管职责，化解各类风险，有效遏制重特大事故发生。加强食品药品安全监管，推进食品药品安全社会共治，积极创建广西“食品安全城市”。加强价格监管，规范市场价格行为。加强应急能力建设，完善城乡综合防灾减灾救灾体系。贯彻落实党的民族政策，促进民族团结进步。积极支持国防和军队建设发展，做好新形势下的双拥工作。

三、切实加强政府自身建设

各位代表！

习近平总书记在山东菏泽考察时曾给市、县干部念过一副对联：“得一官不荣，失一官不辱，勿道一官无用，地方全靠一官；穿百姓之衣，吃百姓之饭，莫以百姓可欺，自己也是百姓。”告诫我们要正确处理好“官”与“民”的关系，做到权为民所用、情为民所系、利为民所谋。站在新的历史起点，面对全市人民的新期待，我们必须对党和人民事业高度负责，以敢于担当的精神、求真务实的作风、真挚为民的情怀，加快建设人民满意的法治政府和服务型政府，真正做到为官一任、造福一方。

（一）增强法治意识，坚持依法行政。坚持在党的领导下，在法治的轨道上行使各项权力、开展各项工作，做到法定职责必须为、法无授权不可为。发挥政府法律顾问团的作用，为政府依法行政当好参谋。完善依法决策机制，政府的重大行政决策都要经过公众参与、专家论证、风险评估、合法性审查和集体讨论决定。全面推行清单制度，推进政府工作部门依法全面履行职责。深化政务公开，落实国务院关于加强政务舆情回应的工作要求，让权力在阳光下运行，切实维护政府公信力。依法接受人大及其常委会的法律监督和工作监督，自觉接受人民政协的民主监督，接受社会和舆论监督，防止滥权失责。

（二）狠抓工作落实，提高政府执行力。政府的重要职责就是按照市委作出的决策部署，狠抓工作落实，推进经济社会向前发展。我们要深入开展“两学一做”学习教育，自觉践行“三严三实”要求，秉持“功成不必在我、功成必定有我”的理念，敢于担当，善作善成，把工作责任扛在肩上，把心思花在谋划北海发展上，把精力用到增进群众福祉上，确保完成各项决策部署和工作任务。加强政府机关作风建设，重点整治“慵懒散拖”、为官不为等现象。继续推行定人员、定职责、定时间、定进度的“四定”办法，推进项目建设和重点工作落实，突出抓好“四大会战”的组织实施。强化督查问责，加强对项目建设和重点工作落实情况逐项督查，把各项工作不折不扣执行到位、落到实处。

（三）优化政务服务，提升行政效率。严格落实政务服务“三集中”和“三项制度”，大力优化政务服务环境，提高服务质量。进一步优化行政审批流程，压缩审批时限，特别是在项目推进、征地拆迁、招商引资、解决群众“急症难症”等方面，要做到靠前主动服务，破除工作“瓶颈”，保持环节顺畅，实现优质高效。大力推行“互联网+”政务服务，加快建设网上办事大厅，变“群众跑腿”为“信息跑路”、变“企业四处找”为“部门协同办”。加快完善基层政务服务中心，把普遍性的服务事项统一纳入乡镇（街道）政务服务中心窗口集中办理。

（四）严守纪律规矩，坚持廉洁从政。严格落实从严治党“两个责任”，认真履行“一岗双责”，深入推进政府系统的党风廉政建设和反腐败工作。坚持把纪律和规矩挺在前面，贯彻执行中国共产党廉洁自律准则、纪律处分条例和问责条例，扎实推进惩治和预防腐败体系建设。严格执行中央八项规定、自治区“十个严禁”和各项廉洁从政纪律，严肃查处发生在群众身边的“四风”和腐败问题。加强廉政教育，强化行政监察和审计监督，重点对公共资金、公共资源、国有资产和重大项目进行监管，促进政府系统廉政建设与业务工作深度融合，做到行政用权之手施展到哪里、执纪问责之剑就悬在哪里，着力营造不敢腐、不能腐、不想腐的政治生态，努力建设廉洁政府。

各位代表！

潮起海天阔，扬帆正当时。蓝图已经绘就，奋斗才能赢得未来。让我们更加紧密团结在以习近平同志为总书记的党中央周围，在自治区党委、政府和市委的正确领导下，凝心聚力、乘胜前进，为在广西率先全面建成小康社会、把北海建设成为充满生机与活力的创新型开放城市而努力奋斗！
